# Multichannel Polysomnographic Sensor Fusion for Automatic Sleep Stage Classification

**DOI:** 10.3390/s26196011

**Published:** 2026-09-23

**Authors:** Simone Mari, Lorenzo Di Vaira, Giovanni Bucci, Fabrizio Ciancetta, Edoardo Fiorucci

**Affiliations:** Dipartimento di Ingegneria Industriale e dell’Informazione e dell’Economia, Università degli Studi dell’Aquila, 67100 L’Aquila, Italy; lorenzo.divaira@student.univaq.it (L.D.V.); giovanni.bucci@univaq.it (G.B.); fabrizio.ciancetta@univaq.it (F.C.); edoardo.fiorucci@univaq.it (E.F.)

**Keywords:** polysomnography, sleep stage classification, sensor fusion, EEG, EOG, EMG, STFT, deep learning, LSTM, edge artificial intelligence

## Abstract

Automatic sleep stage classification can reduce the burden of manual polysomnographic scoring, but reliable models must account for heterogeneous physiological signals, temporal dependencies, and severe class imbalance. This study presents a dual-stream deep learning framework for five-class sleep staging from 30 s polysomnographic segments using two EEG signals, one EOG signal, and one submental EMG signal. Time-domain features are extracted through one-dimensional convolutions, while complementary time–frequency information is obtained from short-time Fourier transform spectrograms processed by a two-dimensional convolutional branch. The resulting features are fused and analyzed over a five-segment context window using a bidirectional long short-term memory network. Prolonged wake periods are censored only during training to reduce majority-class dominance without altering the evaluation distribution. Subject-wise five-fold cross-validation across 78 subjects yielded a mean accuracy of 91.5%, a balanced accuracy of 78.5%, a macro-F1 score of 77.4%, and a Cohen’s kappa of 0.829. Controlled architectural and channel-ablation experiments further quantified the contributions of temporal context, time–frequency processing, wake censoring, and the individual PSG signals. A lightweight time-domain-only variant achieved 88.8% accuracy, 72.7% balanced accuracy, a macro-F1 score of 73.8%, and a Cohen’s kappa of 0.779. These findings support the use of multimodal time–frequency fusion for robust automatic sleep staging while highlighting the trade-off between classification performance and model complexity.

## 1. Introduction

Sleep staging is traditionally performed by trained experts who inspect polysomnographic (PSG) recordings and assign each 30 s scoring segment to wakefulness (W), non-rapid eye movement sleep stages N1, N2, and N3, or rapid eye movement (REM) sleep according to standardized scoring rules [1,2,3]. N1 and N2 are commonly associated with lighter NREM sleep, whereas N3 corresponds to deep or slow-wave sleep. Although this procedure is clinically established, manual scoring is time-consuming and subject to inter-scorer variability, particularly for transitional stages such as N1 [3,4,5]. These limitations have motivated the development of automatic sleep-stage classification methods based on machine learning and deep learning.

Early automatic approaches mainly relied on hand-crafted features and time–frequency analysis of electroencephalographic (EEG), electrooculographic (EOG), and electromyographic (EMG) signals [6,7]. Subsequent deep-learning methods progressively introduced raw-signal convolutional models, multimodal PSG architectures, recurrent sequence models, attention mechanisms, and transformer-based temporal modeling [8,9,10,11,12,13,14,15,16]. Large-scale end-to-end systems have further highlighted the importance of robust architectures, temporal context, and heterogeneous physiological signals for automatic sleep staging [17,18].

The multichannel nature of PSG is particularly relevant because different sensors capture complementary physiological phenomena. Figure 1 illustrates the Sleep-EDF acquisition setting considered in this study. Two bipolar EEG signals, Fpz–Cz and Pz–Oz, provide information on cortical activity; a horizontal EOG signal captures eye-movement-related activity, which is particularly informative for REM sleep and a chin EMG signal provides information on submental muscle tone, which generally decreases during sleep and is strongly reduced during REM sleep.

Automatic classification from these signals remains challenging for several reasons. First, EEG, EOG, and EMG describe different physiological processes and must, therefore, be combined effectively within a common model. Second, sleep stages are characterized by both short-duration waveform patterns and broader spectral characteristics, requiring the simultaneous analysis of temporal and time–frequency information. Third, neighboring sleep segments are not independent because sleep evolves through structured temporal transitions. Finally, the strong imbalance among sleep stages can bias model optimization and make overall accuracy alone insufficient for performance assessment. Accordingly, the aim of this study is to investigate how multichannel PSG information, time-domain and time–frequency feature extraction, temporal context, and training-only class-imbalance handling can be combined within a unified framework for subject-independent five-class sleep-stage classification.

The main contributions of this study are as follows: (i) a dual-stream architecture that jointly processes two EEG signals, one EOG signal, and one EMG signal by combining time-domain convolutional feature extraction with explicit STFT-based time–frequency analysis; (ii) bidirectional temporal modeling over a five-segment context window to incorporate local sleep-stage dependencies; (iii) controlled architectural and signal-ablation experiments that quantify the contributions of the STFT–Conv2D branch, temporal context, the BiLSTM module, training-only wake censoring, and the individual PSG signals and (iv) a subject-wise cross-validation evaluation including aggregate and class-wise metrics, three recording-selection conventions, and comparison with a lightweight time-domain-only model to characterize the trade-off between classification performance and model complexity.

## 2. Materials and Methods

### 2.1. Dataset and Sensor Channels

Experiments were conducted on PSG recordings derived from the public Sleep-EDF Expanded database [19,20]. Each recording contains continuous physiological signals and expert hypnogram annotations. The final experiments used 153 PSG recording files, which corresponded to 78 unique subject identifiers after grouping recordings by the first five characters of the Sleep-EDF subject code. The classification target follows the common five-stage setting: W, N1, N2, N3, and REM. Stages 3 and 4 in the original Rechtschaffen–Kales convention were merged into N3 to align the labels with the modern five-class formulation [2].

The final pipeline relies on four synchronized polysomnographic signals, including two EEG signals, one horizontal EOG signal, and one submental EMG-derived muscle-tone signal. This configuration reflects standard sleep-scoring practice, as EEG activity provides information on cortical rhythms, EOG activity supports the identification of eye movements, and submental EMG activity contributes information on muscle-tone changes, particularly during REM sleep.

In the Sleep-EDF sleep-cassette subset, the EEG and EOG signals are provided as 100 Hz waveforms, whereas the submental EMG channel is available as a low-rate rectified and smoothed muscle-tone envelope. During preprocessing with MNE-Python (version 1.11.0), the EMG envelope was resampled onto the common 100 Hz temporal grid to obtain temporally aligned multichannel tensors. This operation was performed exclusively for temporal alignment and tensor construction; it does not increase the temporal resolution of the original EMG envelope or introduce additional physiological information. The resampled channel was, therefore, treated as a low-frequency representation of submental muscle-tone variation rather than as a raw high-frequency EMG signal. The input components and their corresponding roles in the proposed pipeline are summarized in Table 1.

### 2.2. Pre-Processing and Segment Construction

The PSG and hypnogram EDF files were loaded as paired recordings with MNE-Python [21], and the expert annotations were aligned to the continuous physiological signals before segmentation. Only labeled sleep-stage intervals were retained for supervised learning. Annotation labels were mapped to five classes: W, N1, N2, N3, and REM; original stage 3 and stage 4 labels were merged into N3. Movement and unknown labels were excluded from the supervised set.

The selected EEG and EOG waveforms were low-pass filtered at 30 Hz before segmentation. This preprocessing step attenuated high-frequency components outside the frequency range most relevant for sleep-stage characterization, while preserving the conventional EEG bands used in sleep analysis. The submental EMG channel was treated as a muscle-tone envelope rather than as a raw high-frequency EMG waveform. After temporal alignment to the common segment grid, it was used as a low-frequency representation of submental muscle tone. No explicit high-pass or notch stage was added in the final pipeline. No censoring was applied at this stage to validation or test recordings.

Each recording was segmented into non-overlapping 30 s scoring segments. For a segment-level sample X, the final tensor has shape $N_{\mathrm{ch}}\times N_{s}=4\times 3000$, corresponding to four temporally aligned signals on a common 100 Hz temporal grid. For EEG and EOG, the 3000 samples correspond to the original 30 s waveforms sampled at 100 Hz. For the EMG channel, they correspond to the temporally aligned representation of the original low-rate EMG envelope, not to 3000 independent samples of raw EMG activity. A local z-score normalization was applied channel-wise within each segment after filtering and, for the training generator only, after wake censoring:

(1) $${\tilde{x}}_{\mathrm{ch}}\left[n\right]=\frac{x_{\mathrm{ch}}\left[n\right]-\mu_{\mathrm{ch}}}{\sigma_{\mathrm{ch}}+\epsilon },$$

where $\mu_{\mathrm{ch}}$ and $\sigma_{\mathrm{ch}}$ are the mean and standard deviation of channel ch within the segment, and ϵ is a small numerical constant. This operation reduces amplitude variability due to electrode impedance or inter-recording scaling differences.

To include local temporal context, the generator constructs a sliding window around each target segment:

(2) $$X_{\mathrm{win}}\left[i\right]=\left\{X_{i-2},X_{i-1},X_{i},X_{i+1},X_{i+2}\right\},\;y_{\mathrm{center}}=y_{i}.$$

The resulting input has shape 5×4×3000, corresponding to 2.5 min of PSG context. Edge padding replicates boundary segments so that no segments are discarded at the beginning or end of a recording.

### 2.3. Training-Only Wake Censor

PSG recordings often include long wake periods before sleep onset and after final awakening. This issue is an instance of the broader class-imbalance problem in deep learning, where majority classes can dominate the optimization objective and reduce sensitivity to minority classes [22]. If these wake tails dominate the training set, the optimizer may learn a biased decision boundary that favors W and N2, while under-emphasizing rarer stages such as N1 and N3. To reduce this effect, a wake censor was applied only during training, before z-score normalization and before construction of the five-segment context windows.

The censor operates on each training recording independently. A counter is incremented whenever the current label is W and reset as soon as a non-W label is encountered. The first 60 consecutive W segments of each wake run are retained, corresponding to 30 min of continuous wakefulness. Subsequent W segments in the same run are discarded until the next non-W segment resets the counter. Formally, for a label sequence $\left\{y_{i}\right\}$, the retention mask is updated according to the wake-run length $r_{i}$, and a W segment is retained only if $r_{i}\leq T_{W}$, with $T_{W}=60$.

Validation and test recordings were not censored, cropped, class-weighted, or rebalanced with this procedure. This choice prevents evaluation leakage: the model is optimized on a less wake-dominated training distribution, but it is validated and tested on the natural distribution expected during real PSG scoring.

Importantly, the wake-tail censoring procedure is not a rule-based wakefulness classifier and does not use the physiological signals to determine whether a segment belongs to W or non-W sleep. It operates only on the expert-provided training labels to limit excessively long runs of wakefulness before model optimization. During validation and testing, no such rule is applied: W, N1, N2, N3, and REM are all predicted jointly by the same five-class softmax classifier.

### 2.4. Dual-Stream Time–Frequency Architecture

The proposed architecture contains two complementary feature extractors applied to each 30 s segment. The first branch processes the aligned time-domain channel representations through 1D convolutions. The second branch converts each channel into a time–frequency representation using the STFT and processes the resulting spectrograms through two-dimensional (2D) convolutions. Short-time spectral representations are a classical tool for non-stationary signal analysis and have also been used in previous automatic sleep-staging pipelines [6,23]. The two branch feature extractors are applied independently to each segment of the five-segment context window using a time-distributed implementation. Their outputs are then concatenated at the segment level, producing a five-step feature sequence that is processed by the BiLSTM layer.

The STFT branch computes

(3) $$S_{\log}\left(t,f,\mathrm{ch}\right)=\log\left(1+\left|\mathrm{STFT}\left(x_{\mathrm{ch}}\right)\left(t,f\right)\right|\right),$$

with a frame length of 256 samples and a frame step of 64 samples, corresponding to 2.56 s windows and 0.64 s hops at $f_{s}=100\;\mathrm{Hz}$. The Hann analysis window was used. For each 30 s segment, the transform produced 43 time frames and 129 frequency bins per channel; the tensor was then transposed to a spectrogram-like representation with shape 43×129×4, where the last dimension indexes the PSG channels. Since the transform is implemented as a neural-network layer, the spectrograms are generated on the graphics processing unit during training and inference rather than being precomputed and stored on disk.

### 2.5. Model Specification

The 1D branch uses two convolutional blocks. The first block contains 64 filters with kernel size 50, corresponding to approximately 0.5 s at $f_{s}=100\;\mathrm{Hz}$, followed by max pooling. The second block contains 128 filters with kernel size 8, followed by max pooling and global average pooling. The 2D branch applies the STFT transform, two Conv2D blocks with 32 and 64 filters, respectively, and global average pooling. The fused feature vector is passed to a BiLSTM layer with 64 units and a dropout layer before the final five-class softmax output. The recurrent component is motivated by the ability of LSTM networks to model temporal dependencies in sequential data [24]. The main components of the final model are summarized in Table 2. An overview of the proposed dual-stream architecture is shown in Figure 2.

### 2.6. Lightweight 1D Comparison Model

A compact 1D model was implemented to quantify the value of explicit STFT information. Compact sleep-staging architectures are relevant for wearable and edge-oriented applications, where model size and inference cost can be as important as aggregate accuracy [25]. This version preserves the same preprocessing, four-channel input, five-segment sliding window, time-distributed feature extraction, BiLSTM temporal layer, and training-only wake censor. The only architectural difference is the removal of the STFT and Conv2D branch. This isolates the performance contribution of the frequency-domain stream and provides an estimate of the trade-off between diagnostic performance and computational complexity.

### 2.7. Patient-Independent Split and Training Protocol

The 153 PSG recording files, corresponding to 78 unique subjects, were evaluated using subject-wise five-fold cross-validation. Because the Sleep-EDF naming convention may include more than one recording per subject, subject identity was extracted from the first five characters of each file identifier, and all recordings belonging to the same subject were always assigned to the same partition. At each cross-validation iteration, one mutually exclusive subject fold was held out for testing, while training and validation were performed using only subjects outside the corresponding test fold. Consequently, no subject appeared simultaneously in the training, validation, and test sets.

The held-out test subjects intentionally changed across the five cross-validation iterations, so that each subject contributed to the independent test evaluation exactly once. This design was adopted to assess subject-independent generalization across the entire cohort and to avoid conditioning the reported performance on a single fixed group of test subjects. It also allows the variability associated with test-subject selection to be reflected in the final performance estimates.

The complete dataset contained 414,961 labeled 30 s segments before training-only wake censoring. Because the subjects assigned to the training, validation, and test sets varied across the five cross-validation iterations, the number of segments in each subset also varied slightly from fold to fold. Table 3 therefore reports the exact number of training, validation, and test segments for each cross-validation iteration. For the training set, both the number of segments before wake censoring and the number actually retained for model optimization are reported. Validation and test sets were not censored.

The fold-specific counts also clarify the effect of the training-only wake censor on the data distribution. After censoring, the training sets contained between 115,775 and 118,410 segments, and were, therefore, still substantially larger than the corresponding validation and test sets. Moreover, wakefulness represented approximately 23–24% of the retained training samples, whereas N2 was the most represented training class. By contrast, validation and test recordings retained their natural, uncensored distribution, in which wakefulness was more frequent. This difference was intentional, since class-imbalance mitigation was applied only during model optimization and not during performance evaluation.

TensorFlow was used for model development and training [26]. To reduce memory requirements and improve scalability, PSG recordings were accessed through a streaming data pipeline rather than being fully loaded into random-access memory. Shuffling was applied only to the training data, whereas validation and test data were processed in their original chronological order to preserve the evaluation protocol.

The dual-stream model was trained for a maximum of seven training cycles with the Adam optimizer [27], an initial learning rate of $10^{-3}$, and sparse categorical cross-entropy. Model selection was based on validation loss. Training was regularized through validation-based monitoring. The model parameters corresponding to the lowest validation loss were retained, and the learning rate was adaptively reduced when the validation loss stopped improving, thereby limiting overfitting and stabilizing convergence. A checkpoint saved the best model according to validation loss. Within each cross-validation iteration, the held-out test fold was evaluated only after model selection based on the corresponding validation data. The final performance measures were computed separately for each held-out fold and subsequently summarized as mean and standard deviation across the five iterations.

### 2.8. Evaluation Metrics

All reported metrics were computed at the 30 s segment level, using the expert annotation of the central segment as the reference label for each five-segment context window. The main evaluation metrics were overall accuracy, balanced accuracy, and Cohen’s kappa, together with macro-F1 and class-wise precision, recall, and F1-score.

Overall accuracy was defined as the ratio between the number of correctly classified 30 s test segments and the total number of 30 s test segments:

(4) $$\mathrm{Acc}.=\frac{N_{\mathrm{correct}}}{N_{\mathrm{test}}},$$

where $N_{\mathrm{correct}}$ is the number of test segments for which the predicted sleep stage matched the expert annotation, and $N_{\mathrm{test}}$ is the total number of evaluated test segments.

Balanced accuracy was used to account for the uneven distribution of sleep stages [28] and was computed as the unweighted mean of the class-wise recalls:

(5) $$\mathrm{Bal}.\;\mathrm{Acc}.=\frac{1}{C}\sum_{c=1}^{C}\frac{{\mathrm{TP}}_{c}}{{\mathrm{TP}}_{c}+{\mathrm{FN}}_{c}},$$

where C=5 is the number of sleep-stage classes, and ${\mathrm{TP}}_{c}$ and ${\mathrm{FN}}_{c}$ denote the true positives and false negatives for class *c*, respectively.

Class-wise precision was defined as the proportion of segments predicted as class *c* that were correctly assigned to that class:

(6) $${\mathrm{Precision}}_{c}=\frac{{\mathrm{TP}}_{c}}{{\mathrm{TP}}_{c}+{\mathrm{FP}}_{c}},$$

where ${\mathrm{FP}}_{c}$ denotes the number of false positives for class *c*.

Class-wise recall, also referred to as sensitivity, was defined as the proportion of expert-labeled segments belonging to class *c* that were correctly identified:

(7) $${\mathrm{Recall}}_{c}=\frac{{\mathrm{TP}}_{c}}{{\mathrm{TP}}_{c}+{\mathrm{FN}}_{c}}.$$

The class-wise F1-score was computed as the harmonic mean of precision and recall:

(8) $$F1_{c}=2\;\frac{{\mathrm{Precision}}_{c}\;{\mathrm{Recall}}_{c}}{{\mathrm{Precision}}_{c}+{\mathrm{Recall}}_{c}}.$$

This metric provides a combined assessment of false-positive and false-negative errors for each sleep stage.

The macro-F1 score was calculated as the unweighted mean of the five class-wise F1-scores:

(9) $$\text{Macro-F}1=\frac{1}{C}\sum_{c=1}^{C}F1_{c}.$$

Because each class contributes equally to the average, macro-F1 provides an assessment that is less dominated by the most frequent sleep stages.

Cohen’s kappa was used to quantify agreement between the model predictions and expert annotations while correcting for chance agreement:

(10) $$\kappa_{\mathrm{Cohen}}=\frac{p_{o}-p_{e}}{1-p_{e}},$$

where $p_{o}$ is the observed agreement and $p_{e}$ is the expected agreement under the empirical class marginals [29].

Because the sleep-stage distribution is imbalanced, overall accuracy was reported primarily as a conventional aggregate measure and was complemented by balanced accuracy and macro-F1. These aggregate metrics are directly consistent with the corresponding class-wise measures: balanced accuracy is the unweighted mean of the class-wise recalls, whereas macro-F1 is the unweighted mean of the class-wise F1-scores. Class-wise precision is additionally reported to characterize false-positive behavior for each stage. Therefore, performance is not assessed from overall accuracy or class-wise precision alone, but from complementary global and class-balanced measures.

Standard classification metrics and confusion matrices were computed using scikit-learn [30]. Confusion matrices were reported both as raw counts and as row-normalized percentages, so that each row represents the recall distribution for one expert-labeled sleep stage.

## 3. Results

### 3.1. Overall Classification Performance

The dual-stream model achieved the best test-set performance, with 91.5% overall accuracy, 78.5% balanced accuracy, and $\kappa_{\mathrm{Cohen}}$ = 0.829. The lightweight 1D variant reached 88.8% accuracy, 72.7% balanced accuracy, and $\kappa_{\mathrm{Cohen}}$ = 0.779. The improvement of the dual-stream model indicates that explicit spectrogram processing provides information not fully recovered by temporal convolutions alone. A quantitative comparison of the different model configurations is reported in Table 4.

### 3.2. Five-Fold Cross-Validation Performance

To obtain a more robust estimate of subject-independent generalization, the proposed dual-stream model was evaluated using subject-wise five-fold cross-validation. The 78 subjects were divided into five mutually exclusive folds, ensuring that all recordings belonging to the same subject were assigned to the same fold. At each cross-validation iteration, one fold was held out for testing, while model training, validation, and checkpoint selection were performed using only subjects outside the corresponding test fold. The preprocessing procedure, five-segment temporal context, dual-stream architecture, optimization settings, and training-only wake-censoring strategy were applied independently in each iteration.

The evaluation metrics were first computed separately on each held-out test fold and then summarized across the five cross-validation iterations. Table 5 reports the mean and standard deviation of the global performance measures and the class-wise precision, recall, and F1-score.

The cross-validation analysis yielded a mean overall accuracy of 91.5%, a mean balanced accuracy of 78.5%, a mean macro-F1 score of 77.4%, and a mean Cohen’s kappa of 0.829. The similar values of balanced accuracy and macro-F1 indicate that these two aggregate measures provide a consistent assessment of performance across the five imbalanced sleep-stage classes.

At the class level, wakefulness showed the highest performance, with a mean precision of 99.2%, a mean recall of 96.9%, and a mean F1-score of 98.0%. The model also achieved mean F1-scores of 83.4% for non-rapid eye movement stage 2 sleep, 82.6% for rapid eye movement sleep, and 75.9% for non-rapid eye movement stage 3 sleep. The mean recall for non-rapid eye movement stage 3 sleep reached 75.0%, indicating that the lower value observed in the original single-split experiment was not representative of the average performance across the held-out subject folds.

Non-rapid eye movement stage 1 sleep remained the most challenging class, with a mean precision of 45.2%, a mean recall of 49.0%, and a mean F1-score of 47.1%. The comparatively large standard deviation of its recall also indicates greater variability across the held-out subject groups in the recognition of this transitional sleep stage.

To evaluate the sensitivity of the results to the temporal selection of the recordings, the held-out test predictions were additionally assessed under three evaluation conventions: complete uncropped recordings, the annotated in-bed interval extended by 30 min before and after the in-bed period, and the in-bed interval only. For each five-segment input window, the prediction referred to the central 30 s segment. The number of evaluated test windows, therefore, corresponds to the number of central segments retained under each convention across the held-out test folds.

Table 6 reports the number of evaluated test windows, overall accuracy, and macro-F1 obtained under the three conventions.

Overall accuracy decreased from 91.48% with uncropped recordings to 82.57% with the in-bed interval extended by 30 min and to 81.30% with the in-bed-only convention. In contrast, macro-F1 remained substantially stable, decreasing only from 77.36% to 77.03% and 76.92%, respectively. The difference between the trends in accuracy and macro-F1 indicates that the higher accuracy obtained with uncropped recordings was influenced by the larger proportion of wakefulness segments in the complete recordings. Because macro-F1 assigns equal importance to all five sleep stages, its limited variation shows that the average class-balanced performance of the model was considerably less sensitive to the selected temporal convention.

Overall, the subject-wise cross-validation results and the comparison among the three temporal evaluation conventions provide a more robust and transparent characterization of model generalization than an evaluation based on a single held-out subject partition or a single recording-cropping convention.

### 3.3. Architectural Ablation Study

A controlled ablation study was conducted to assess the contribution of the principal components of the proposed dual-stream architecture. Four variants were independently trained by removing the training-only wake-tail censoring procedure, the five-segment temporal context, the BiLSTM module, or the STFT–Conv2D branch. The remaining preprocessing, input signals, optimization procedure, and subject-independent data partition were kept unchanged.

Each ablated model was evaluated on the same held-out test subjects. Overall accuracy, balanced accuracy, macro-F1, Cohen’s kappa, and class-wise precision, recall, and F1-score were computed at the 30 s segment level. The contribution of the individual physiological signals was examined separately through a leave-one-signal-out analysis and is therefore not included in the present architectural ablation study.

Table 7 reports the global performance of the four ablated configurations.

Table 8 reports the class-wise results of the same ablated configurations.

Among the ablated configurations, removing temporal context produced the lowest global performance, with an accuracy of 87.65%, a balanced accuracy of 70.92%, a macro-F1 score of 70.08%, and a Cohen’s kappa of 0.7533. The largest class-specific deterioration was observed for non-rapid eye movement stage 1 sleep, whose recall decreased to 25.24% and whose F1-score decreased to 33.49%. Rapid eye movement sleep was also affected, reaching an F1-score of 70.14%. These findings indicate that neighboring sleep segments provide relevant information for the recognition of transitional and temporally dependent stages.

Removal of the BiLSTM module resulted in an accuracy of 88.60%, a balanced accuracy of 71.80%, a macro-F1 score of 73.12%, and a Cohen’s kappa of 0.7735. Although the recall for non-rapid eye movement stage 2 sleep remained high at 87.10%, the F1-score for non-rapid eye movement stage 1 sleep was limited to 41.49%. The reduction in performance indicates that temporal context alone is not sufficient and that recurrent modeling contributes to the interpretation of the five-segment sequence.

When the STFT–Conv2D branch was removed, the time-domain-only model achieved an accuracy of 88.93%, a balanced accuracy of 72.57%, a macro-F1 score of 74.40%, and a Cohen’s kappa of 0.7791. This was the highest macro-F1 score among the four ablated configurations. However, the recall and F1-score for non-rapid eye movement stage 3 sleep decreased to 63.40% and 69.99%, respectively. This result suggests that the explicit time–frequency representation may be particularly useful for stages characterized by distinctive spectral patterns, including slow-wave sleep.

Removing training-only wake-tail censoring yielded the highest accuracy among the ablated configurations, equal to 89.19%, but resulted in a lower macro-F1 score of 72.60%. Wakefulness achieved an F1-score of 97.47%, whereas the recall and F1-score for non-rapid eye movement stage 1 sleep decreased to 28.95% and 39.27%, respectively. The difference between overall accuracy and macro-F1 indicates that retaining all prolonged wakefulness segments favored performance on the majority class without producing a corresponding improvement across all sleep stages.

Overall, non-rapid eye movement stage 1 sleep was the most difficult class in every ablated configuration. The strongest reduction was observed after removing temporal context, followed by the removal of wake-tail censoring and the BiLSTM module. Conversely, removal of the STFT–Conv2D branch had a more pronounced effect on non-rapid eye movement stage 3 sleep. These class-specific patterns support the use of balanced accuracy, macro-F1, and class-wise metrics in addition to overall accuracy when assessing the contribution of individual model components.

### 3.4. PSG Channel Ablation Study

A second controlled ablation study was conducted to assess the contribution of the PSG input channels to classification performance. Three additional variants of the dual-stream model were independently trained by removing the two EEG channels jointly, the EOG channel, or the EMG channel. In each experiment, the remaining architecture, preprocessing procedure, five-segment temporal context, training-only wake-tail censoring strategy, optimization settings, and subject-independent data partition were kept unchanged. Because the two EEG channels were removed together, this experiment evaluates the contribution of the EEG channel pair as a whole rather than the individual contributions of the Fpz–Cz and Pz–Oz channels.

Table 9 reports the global performance of the full dual-stream model and of the three PSG channel ablation configurations.

Table 10 reports the corresponding class-wise precision, recall, and F1-scores.

Removal of both EEG channels produced the largest deterioration in global performance. Accuracy decreased from 91.5% to 85.6%, balanced accuracy from 78.5% to 63.3%, macro-F1 from 77.4% to 65.9%, and Cohen’s kappa from 0.829 to 0.710. This corresponded to reductions of 5.9 percentage points in accuracy, 15.2 percentage points in balanced accuracy, and 11.5 percentage points in macro-F1.

The absence of both EEG channels particularly affected non-rapid eye movement stage 1 sleep, non-rapid eye movement stage 3 sleep, and rapid eye movement sleep. The F1-score for non-rapid eye movement stage 1 sleep decreased from 47.1% to 32.1%, while the F1-score for non-rapid eye movement stage 3 sleep decreased from 75.9% to 58.9%. The F1-score for rapid eye movement sleep also decreased from 82.6% to 68.2%. These results confirm the central role of the EEG channels in capturing cortical rhythms, slow-wave activity, and sleep-stage transitions.

Removal of the EOG channel resulted in an accuracy of 88.2%, a balanced accuracy of 72.3%, a macro-F1 score of 71.9%, and a Cohen’s kappa of 0.762. The most pronounced reductions were observed for non-rapid eye movement stage 1 sleep and rapid eye movement sleep, whose F1-scores decreased to 38.2% and 74.7%, respectively. The decrease in rapid eye movement sleep performance is consistent with the role of the EOG channel in capturing eye-movement-related activity.

The model without the EMG channel retained the highest aggregate performance among the channel-ablation variants, with an accuracy of 88.8%, a balanced accuracy of 73.5%, a macro-F1 score of 74.4%, and a Cohen’s kappa of 0.778. Nevertheless, removal of the EMG channel reduced the F1-score for rapid eye movement sleep from 82.6% to 77.9%. The F1-scores for non-rapid eye movement stages 2 and 3 also decreased to 78.5% and 72.0%, respectively.

Although the EMG channel available in the Sleep-EDF recordings is a low-rate muscle-tone envelope rather than a raw high-frequency EMG waveform, the observed reductions indicate that it provides complementary information for sleep-stage classification. Overall, the PSG channel ablation study shows that the EEG channel pair provides the largest contribution, while the EOG and EMG channels supply additional information that improves the balanced recognition of the five sleep stages.

### 3.5. Confusion-Matrix Analysis

Figure 3 and Figure 4 report the test-set confusion matrices for the proposed dual-stream model and the lightweight 1D variant. Rows correspond to expert labels and columns correspond to model predictions. The color scale is row-normalized so that each row sums to 100%; raw sample counts and row percentages are both printed inside each cell. This representation avoids visually overemphasizing the majority W class and highlights the recall pattern within each sleep stage.

The row-wise recalls of the dual-stream model were 96.9% for W, 49.0% for N1, 85.3% for N2, 75.0% for N3, and 86.1% for REM. The corresponding recalls of the lightweight 1D variant were 97.0%, 39.2%, 86.6%, 68.0%, and 72.7%, respectively. These values show that the aggregate improvement of the dual-stream model was mainly associated with better N1, N3, and REM recognition, while N1 remained the most challenging stage.

### 3.6. Effect of Time–Frequency Fusion

The difference between the dual-stream and lightweight models was 2.7 percentage points in overall accuracy and 0.050 in $\kappa_{\mathrm{Cohen}}$. Because both models used the same four channels, five-segment temporal context, and training-only wake censor, this gap suggests that the STFT–Conv2D branch contributed useful complementary information. The frequency-domain stream may be especially relevant for stages that share similar waveform amplitudes but differ in spectral composition, although this interpretation should be confirmed with additional ablation studies.

### 3.7. Clinical Interpretation

The achieved mean $\kappa_{\mathrm{Cohen}}=0.829$ lies in the range usually interpreted as almost perfect agreement [31]. This value is compatible with clinically meaningful agreement, but comparisons with human inter-scorer reliability should be interpreted cautiously because they depend on the dataset, scorer pool, scoring rules, and evaluation protocol [3]. The lightweight 1D model, with a mean $\kappa_{\mathrm{Cohen}}=0.779$, remained within the substantial-agreement range, suggesting that it may be useful when memory, latency, or energy constraints make real-time STFT computation impractical.

## 4. Discussion

The proposed results support three main observations. First, multichannel PSG sensor fusion appears useful because EEG, EOG, and EMG encode complementary physiological information. The EMG channel may help discriminate REM-related muscle atonia and reduce ambiguity between REM and low-amplitude non-REM transitions. Second, explicit STFT-based features were associated with improved performance compared with a purely 1D architecture under the same contextual configuration. While 1D convolutions can learn filter-like operations, the STFT branch provides a structured time–frequency representation that makes spectral patterns directly accessible to 2D convolutional filters. Third, class-imbalance handling must be separated from evaluation. Applying the wake censor only to the training set reduces wake dominance during optimization without producing artificially favorable validation or test distributions. The high wakefulness precision should, therefore, be interpreted together with its recall and F1-score and with the class-balanced aggregate metrics, rather than as an isolated measure, particularly because the uncensored validation and test recordings preserve the naturally wake-dominated distribution.

To place the proposed framework in the context of previous automatic sleep-staging methods, Table 11 compares its performance with representative approaches evaluated on the Sleep-EDF Expanded v2-2018 dataset under the in-bed-only convention. The in-bed-only results of the proposed model are reported to reduce the influence of the prolonged wake periods present in uncropped recordings and to provide a more conservative comparison with previous studies.

All methods included in Table 11 address the same five-class sleep-staging task, comprising W, N1, N2, N3, and REM.

Within the five-class in-bed-only benchmark reported in Table 11, the proposed dual-stream model achieved an accuracy of 81.3% and a macro-F1 score of 76.9%. These values were higher than those reported for all the representative methods included in the comparison. In particular, XSleepNet2, which showed the strongest performance among the previous approaches, achieved 80.3% accuracy and a 76.4% macro-F1 score. These results indicate that the proposed multichannel time–frequency fusion framework is competitive with established sleep-staging architectures while explicitly integrating complementary EEG, EOG, and EMG information.

The model design is consistent with prior findings that temporal context improves sleep stage classification [11,12]. Unlike approaches relying only on raw single-channel EEG [10], the proposed model integrates four PSG channels and combines time-domain and frequency-domain streams. The comparison with the compact 1D version also makes the computational trade-off explicit: the STFT branch improved accuracy and agreement in this experiment, whereas the 1D version may be better suited for embedded deployment.

This study remains limited to evaluation on the Sleep-EDF Expanded database. Although subject-wise five-fold cross-validation provides a more robust estimate of generalization than a single held-out split, external cross-dataset validation would further strengthen the assessment of model generalizability. In addition, computational cost should be quantified in terms of parameter count, floating-point operations, memory footprint, and inference latency on target edge hardware. Future work should also evaluate subject-level calibration, external datasets, and explainability methods to relate learned features to clinically interpretable PSG patterns.

## 5. Conclusions

This paper presented a dual-stream deep learning pipeline for automatic sleep stage classification based on four-channel PSG sensor fusion. The architecture combines raw time-domain convolutional features, STFT spectrogram features, and five-segment bidirectional temporal modeling. A training-only wake censor reduces the imbalance caused by prolonged wake tails without altering validation or test recordings. The proposed model achieved a mean accuracy of 91.5%, a mean balanced accuracy of 78.5%, a mean macro-F1 score of 77.4%, and a mean Cohen’s kappa of $\kappa_{\mathrm{Cohen}}=0.829$, improving over a lightweight 1D comparison model under the same subject-wise five-fold cross-validation protocol. These findings suggest that time–frequency sensor fusion is a useful design choice for clinically oriented sleep staging, while the compact 1D model remains a practical alternative for resource-constrained applications.

The present study remains limited to evaluation on the Sleep-EDF Expanded database and does not yet include cross-dataset validation or a hardware-level assessment of computational complexity. Future work will, therefore, focus on external validation, subject-level calibration, explainability analyses, and the measurement of parameter count, memory usage, inference latency, and energy consumption on resource-constrained hardware.

Recent developments in clinically oriented AI emphasize that predictive performance should be considered together with interpretability, multimodal information integration, and generalizability. In this context, the present channel-ablation results provide a model-level indication of the complementary contribution of EEG, EOG, and EMG, while future work should investigate segment-level explainability and external validation across datasets and acquisition settings [35]. These aspects are important for supporting the reliable clinical translation of automatic sleep-staging systems.

## Figures and Tables

**Figure 1:**
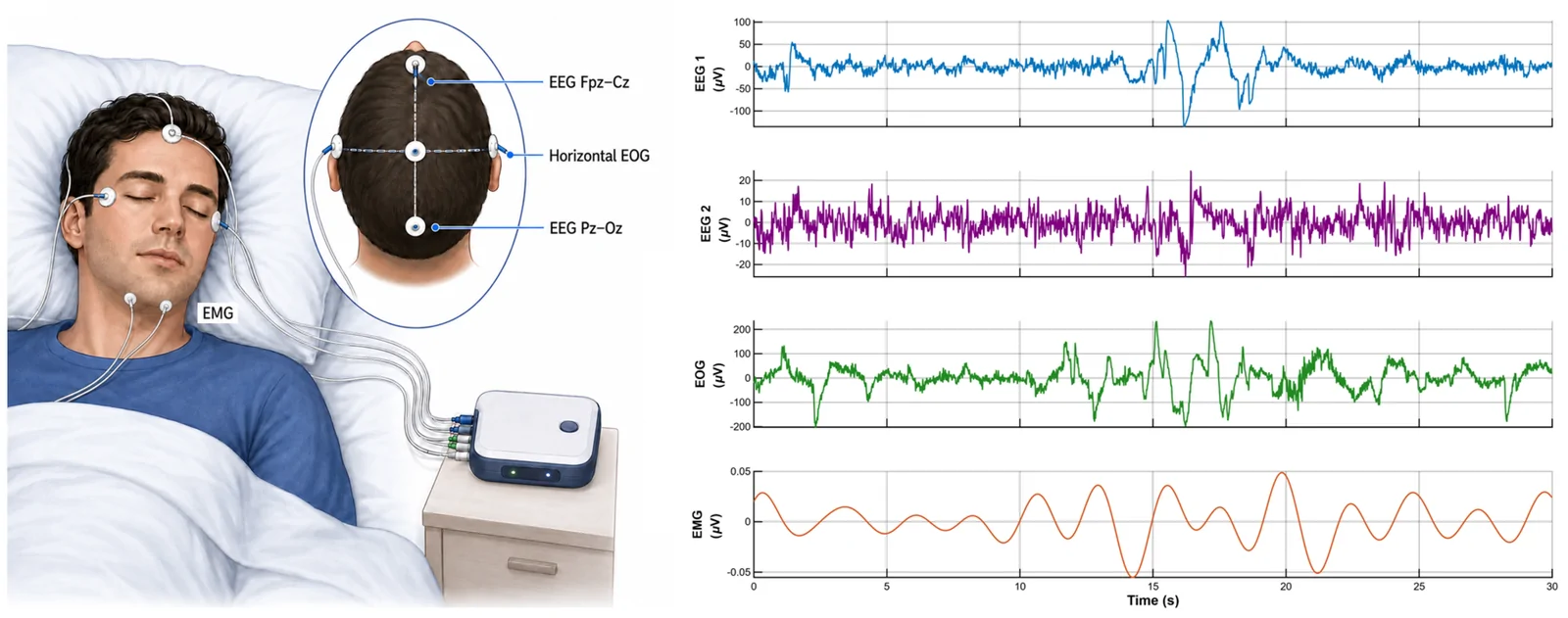
Schematic overview of the Sleep-EDF acquisition setting considered in this study. The four-channel input combines two EEG signals, Fpz–Cz and Pz–Oz, one horizontal EOG signal, and one chin EMG signal. The EMG sensor placement is represented schematically because the public Sleep-EDF documentation does not report subject-specific electrode geometry.

**Figure 2:**
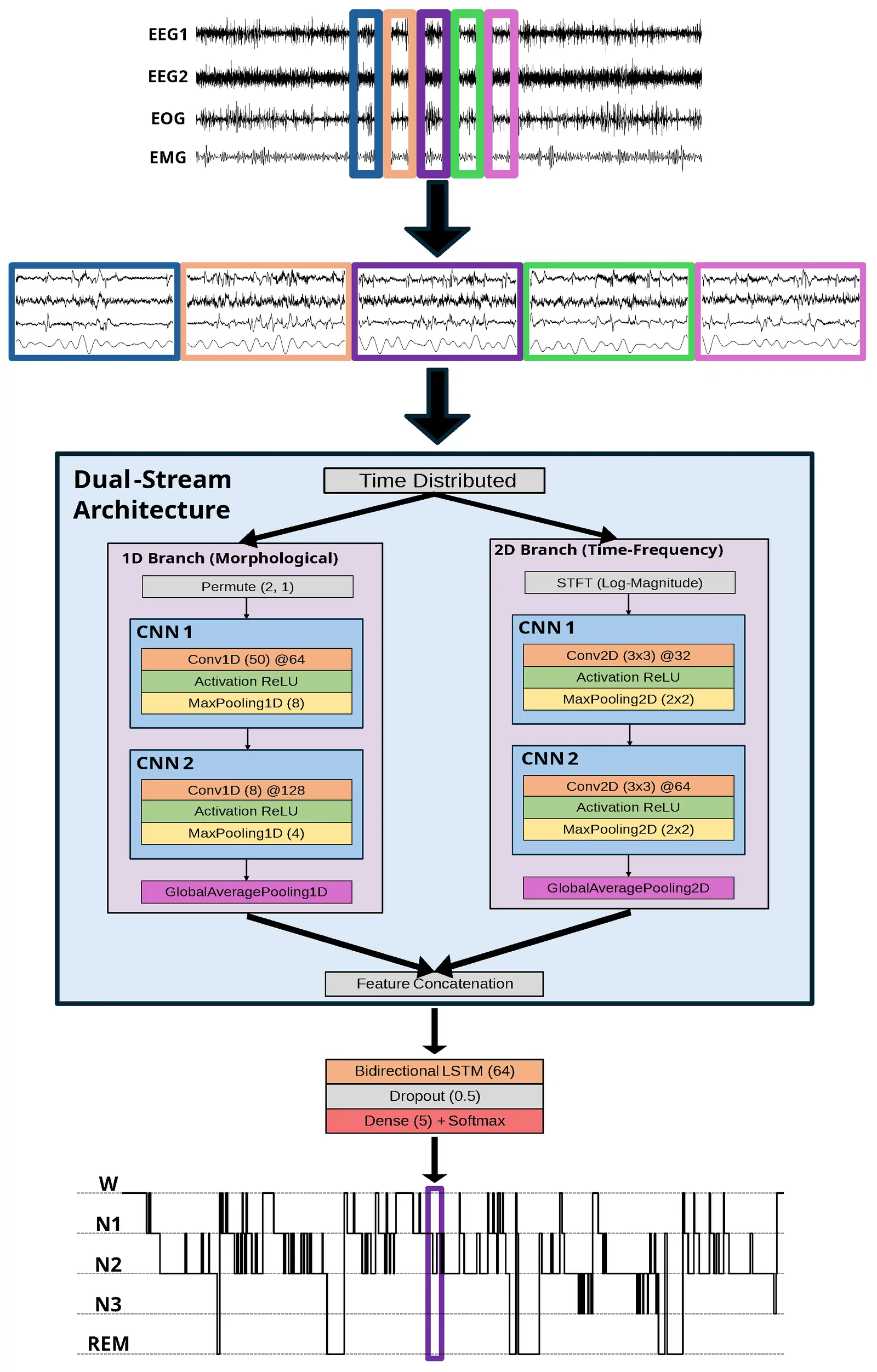
Overview of the proposed dual-stream architecture. Time-domain and STFT-based representations are extracted in parallel and fused before temporal modeling. Colored frames denote consecutive segments, while arrows indicate data flow.

**Figure 3:**
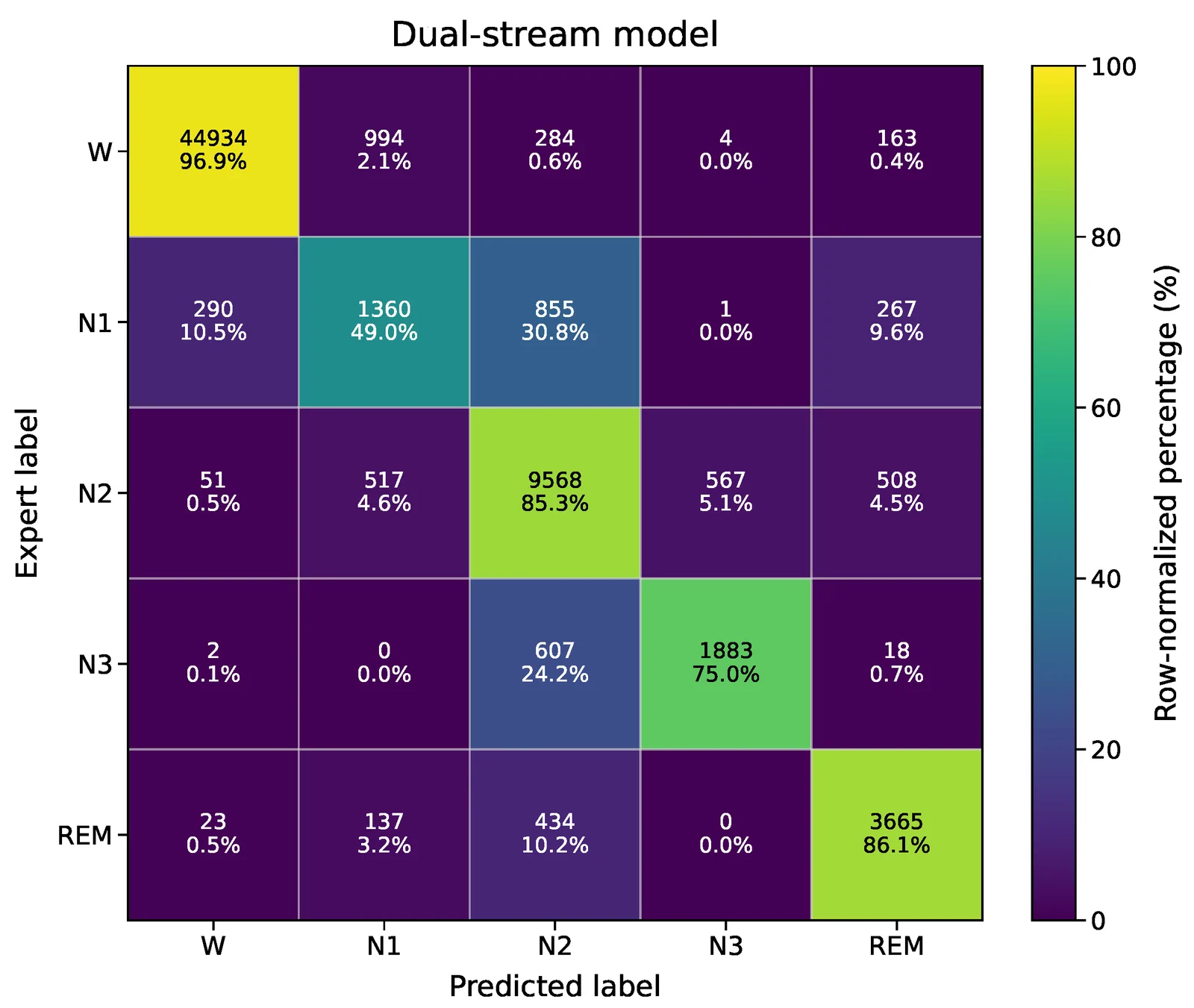
Test-set confusion matrix of the proposed dual-stream model. Rows are expert labels and columns are predicted labels. The color scale reports row-normalized percentages, while cell annotations report raw counts and percentages.

**Figure 4:**
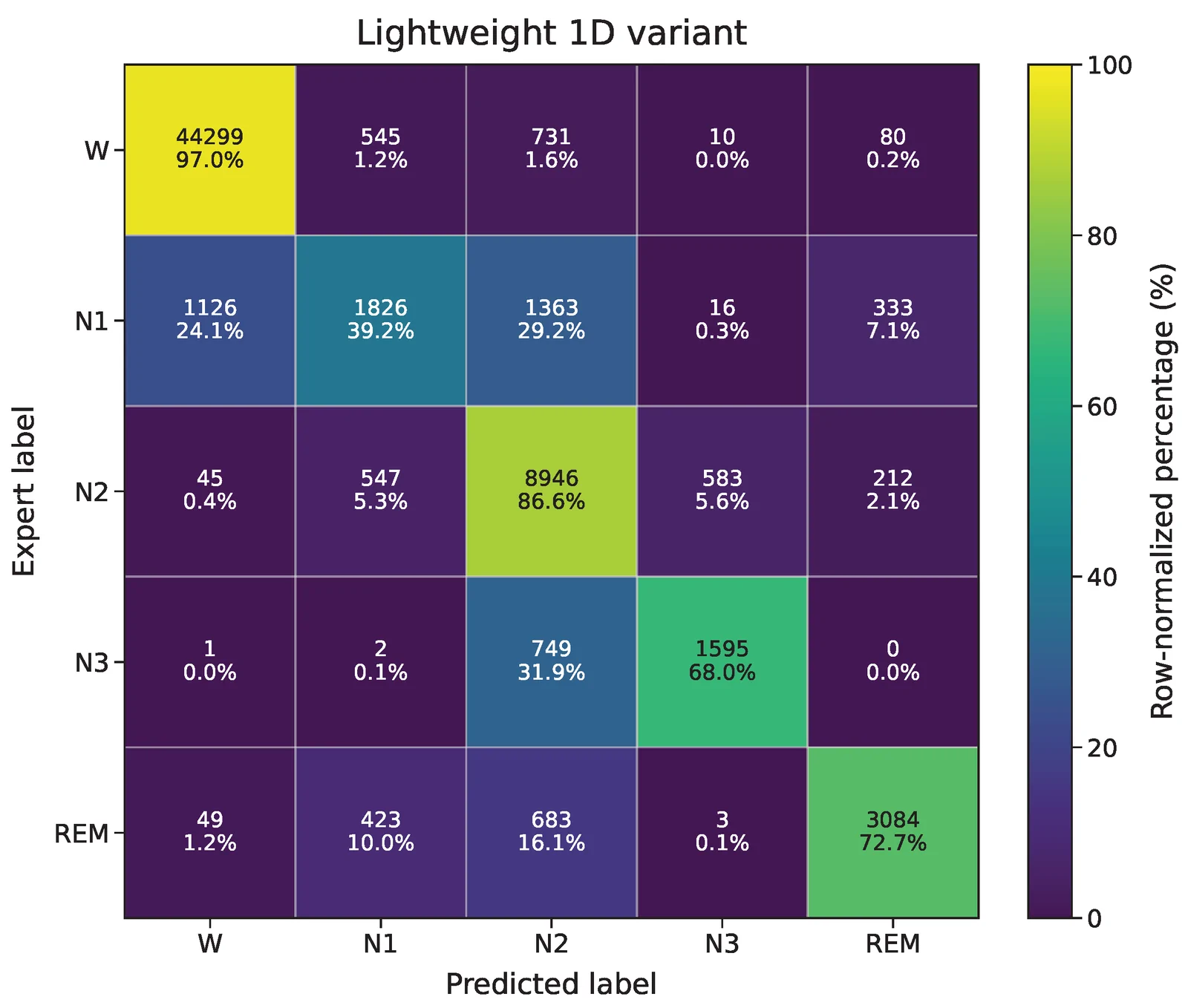
Test-set confusion matrix of the lightweight 1D variant. Rows are expert labels and columns are predicted labels. The color scale reports row-normalized percentages, while cell annotations report raw counts and percentages.

**Table 1:** Input representation used by the proposed pipeline.

Component	Role	Representation
EEG channels	Cortical rhythms, slow waves, spindles, and K-complexes	Two temporal waveforms per 30 s segment
EOG channel	Eye movement information, especially useful for REM and transitions	One temporal waveform per 30 s segment
EMG channel	Muscle-tone information, relevant to REM and wake/sleep separation	One 30 s EMG-envelope signal
Context window	Sequential information around the target segment	$N_{\mathrm{win}}=5$ segments: two previous, one central, two following

**Table 2:** Summary of the final dual-stream model.

Stage	Operation	Purpose
Input	5×4×3000 PSG tensor	Local temporal context around the central segment
Time-domain branch	Conv1D(64, 50), pooling, Conv1D(128, 8), pooling, global average pooling	Learns morphology and transient waveform patterns
Frequency-domain branch	STFT, log magnitude, Conv2D(32), pooling, Conv2D(64), pooling, global average pooling	Learns time–frequency signatures such as alpha, theta, delta activity, and spindles
Fusion	Concatenation of 1D and 2D features	Integrates complementary sensor representations
Temporal model	Bidirectional LSTM with 64 units	Models transitions across five consecutive segments
Classifier	Dropout and Dense softmax layer	Outputs probabilities for W, N1, N2, N3, and REM

**Table 3:** Distribution of 30 s labeled segments across the five subject-wise cross-validation iterations. The complete pre-censor dataset contains 414,961 labeled segments.

Number of Segments in Each Cross-Validation Iteration						
Fold	Training Pre-Censor	Training Used		Validation		Test
1	287,611	118,410		60,218		67,132
2	288,003	117,280		59,707		67,251
3	284,713	115,775		60,407		69,841
4	287,287	116,544		60,072		67,602
5	287,458	118,394		60,066		67,437
**Sleep-stage distribution in each cross-validation iteration**						
**Fold**	**Set**	**W**	**N1**	**N2**	**N3**	**REM**
1	Training	28,732	15,845	47,671	8588	17,574
	Validation	41,121	2904	10,250	1941	4002
	Test	46,379	2773	11,211	2510	4259
2	Training	28,215	14,377	48,438	8938	17,312
	Validation	40,830	2481	10,361	1754	4281
	Test	45,665	4664	10,333	2347	4242
3	Training	27,009	14,524	46,825	9630	17,787
	Validation	41,509	3013	10,563	1735	3587
	Test	47,977	3985	11,744	1674	4461
4	Training	28,040	15,104	46,312	9321	17,767
	Validation	41,246	3123	9592	2246	3865
	Test	45,404	3295	13,228	1472	4203
5	Training	28,933	15,346	47,449	9058	17,608
	Validation	41,748	3006	10,122	1470	3720
	Test	45,688	3170	11,561	2511	4507

**Table 4:** Performance comparison across the main development stages.

Configuration	Input and Architecture	Acc.	Bal. Acc.	$\kappa_{\mathrm{Cohen}}$
Initial baseline	3 channels; single 30 s segment; simple 1D CNN	75.0%	64.5%	0.473
Intermediate model	4 channels; CNN + LSTM; single 30 s segment	86.3%	56.2%	0.719
Wake-optimized model	4 channels; CNN + LSTM; single 30 s segment; wake censor applied during training only	83.0%	66.8%	0.682
Proposed dual-stream model	4 channels; 5-segment context window; 1D branch + STFT–2D branch + BiLSTM	**91.5%**	**78.5%**	**0.829**
Lightweight 1D variant	4 channels; 5-segment context window; 1D CNN + BiLSTM	88.8%	72.7%	0.779

Acc.: accuracy; Bal. acc.: balanced accuracy.

**Table 5:** Five-fold subject-wise cross-validation performance of the proposed dual-stream model.

Global Performance Metrics			
**Metric**	**Mean ± standard deviation**		
Overall accuracy	91.5%±0.7%		
Balanced accuracy	78.5%±2.2%		
Macro-F1	77.4%±1.8%		
Cohen’s κ	0.829±0.012		
**Class-wise performance metrics**			
**Sleep stage**	**Precision**	**Recall**	**F1-score**
W	99.2%±0.9%	96.9%±0.6%	98.0%±0.5%
N1	45.2%±3.1%	49.0%±7.4%	47.1%±3.9%
N2	81.4%±2.6%	85.3%±3.2%	83.4%±1.9%
N3	76.7%±7.1%	75.0%±6.4%	75.9%±3.6%
REM	79.3%±3.6%	86.1%±5.9%	82.6%±3.3%

**Table 6:** Performance of the proposed dual-stream model under the three temporal evaluation conventions.

Evaluation Convention	Evaluated Test Windows	Accuracy	Macro-F1
Uncropped recordings	67,852	91.5%	77.4%
In-bed interval ± 30 min	29,917	82.6%	77.0%
In-bed interval only	27,030	81.3%	76.9%

**Table 7:** Global performance of the full dual-stream model and of the architectural ablation configurations.

Configuration	Accuracy	Balanced Accuracy	Macro-F1	Cohen’s κ
**Full Dual-Stream Model**	**91.5%**	**78.5%**	**77.4%**	**0.829**
Without wake-tail censoring	89.2%	73.6%	72.6%	0.782
Without temporal context	87.7%	70.9%	70.1%	0.753
Without BiLSTM	88.6%	71.8%	73.1%	0.774
Without STFT–Conv2D branch	88.9%	72.6%	74.4%	0.779

**Table 8:** Class-wise performance of the full dual-stream model and of the architectural ablation configurations.

Configuration	Stage	Precision	Recall	F1-Score
**Full dual-stream model**	**W**	**99.2%**	**96.9%**	**98.0%**
**Full dual-stream model**	**N1**	**45.2%**	**49.0%**	**47.1%**
**Full dual-stream model**	**N2**	**81.4%**	**85.3%**	**83.4%**
**Full dual-stream model**	**N3**	**76.7%**	**75.0%**	**75.9%**
**Full dual-stream model**	**REM**	**79.3%**	**86.1%**	**82.6%**
Without wake-tail censoring	W	96.4%	98.5%	97.5%
Without wake-tail censoring	N1	61.0%	29.0%	39.3%
Without wake-tail censoring	N2	77.8%	81.7%	79.7%
Without wake-tail censoring	N3	65.2%	80.3%	72.0%
Without wake-tail censoring	REM	71.2%	78.4%	74.6%
Without temporal context	W	96.5%	97.3%	96.9%
Without temporal context	N1	49.8%	25.2%	33.5%
Without temporal context	N2	72.8%	81.3%	76.8%
Without temporal context	N3	70.1%	76.3%	73.1%
Without temporal context	REM	66.2%	74.6%	70.1%
Without BiLSTM	W	96.5%	97.3%	96.9%
Without BiLSTM	N1	50.9%	32.6%	41.5%
Without BiLSTM	N2	71.5%	87.1%	78.5%
Without BiLSTM	N3	73.6%	70.6%	72.0%
Without BiLSTM	REM	82.1%	71.8%	76.6%
Without STFT–Conv2D branch	W	96.8%	97.3%	97.1%
Without STFT–Conv2D branch	N1	51.7%	43.4%	47.2%
Without STFT–Conv2D branch	N2	73.8%	84.2%	78.7%
Without STFT–Conv2D branch	N3	78.1%	63.4%	70.0%
Without STFT–Conv2D branch	REM	84.2%	74.6%	79.1%

**Table 9:** Global performance of the full dual-stream model and of the PSG channel ablation configurations.

Configuration	Accuracy	Balanced Accuracy	Macro-F1	Cohen’s κ
**Full Dual-Stream Model**	**91.5%**	**78.5%**	**77.4%**	**0.829**
Without both EEG channels	85.6%	63.3%	65.9%	0.710
Without the EOG channel	88.2%	72.3%	71.9%	0.762
Without the EMG channel	88.8%	73.5%	74.4%	0.778

**Table 10:** Class-wise performance of the full dual-stream model and of the PSG channel ablation configurations.

Configuration	Stage	Precision	Recall	F1-Score
**Full dual-stream model**	**W**	**99.2%**	**96.9%**	**98.0%**
**Full dual-stream model**	**N1**	**45.2%**	**49.0%**	**47.1%**
**Full dual-stream model**	**N2**	**81.4%**	**85.3%**	**83.4%**
**Full dual-stream model**	**N3**	**76.7%**	**75.0%**	**75.9%**
**Full dual-stream model**	**REM**	**79.3%**	**86.1%**	**82.6%**
Without both EEG channels	W	94.8%	96.1%	95.4%
Without both EEG channels	N1	40.5%	26.5%	32.1%
Without both EEG channels	N2	66.7%	85.4%	74.9%
Without both EEG channels	N3	74.0%	48.9%	58.9%
Without both EEG channels	REM	79.8%	59.5%	68.2%
Without the EOG channel	W	95.8%	97.9%	96.8%
Without the EOG channel	N1	52.0%	30.2%	38.2%
Without the EOG channel	N2	75.6%	78.4%	77.0%
Without the EOG channel	N3	68.1%	77.7%	72.6%
Without the EOG channel	REM	72.3%	77.1%	74.7%
Without the EMG channel	W	96.8%	97.1%	96.9%
Without the EMG channel	N1	53.5%	41.2%	46.6%
Without the EMG channel	N2	73.7%	84.0%	78.5%
Without the EMG channel	N3	72.0%	71.9%	72.0%
Without the EMG channel	REM	83.0%	73.4%	77.9%

**Table 11:** Comparison with representative sleep-staging methods evaluated on Sleep-EDF Expanded v2-2018 under the in-bed-only convention.

Method	Accuracy	Macro-F1
DeepSleepNet [10]	76.0%	72.2%
SeqSleepNet+ [32]	79.0%	74.6%
DeepSleepNet-Lite [33]	79.0%	75.1%
Naive Fusion [34]	79.1%	75.1%
FCNN+RNN [34]	79.3%	75.1%
XSleepNet2 [34]	80.3%	76.4%
**Proposed dual-stream model**	**81.3%**	**76.9%**

## Data Availability

The Sleep-EDF Expanded database is publicly available through PhysioNet.
